# The Impact of Artificial Intelligence on Lung Cancer Diagnosis and Personalized Treatment

**DOI:** 10.3390/ijms26178472

**Published:** 2025-08-31

**Authors:** Yaman Ayasa, Diyar Alajrami, Mayar Idkedek, Kareem Tahayneh, Firas Abu Akar

**Affiliations:** 1 Faculty of Medicine, Al-Quds University, East Jerusalem 20002, Palestine; yaman.ayasa@students.alquds.edu (Y.A.); diyar.alajrami@students.alquds.edu (D.A.); mayar.idkedek@gmail.com (M.I.); ktahayna@gmail.com (K.T.); 2 Department of General Surgery, Faculty of Medicine, Al-Quds University, East Jerusalem 20002, Palestine; 3 Department of Thoracic Surgery, The Edith Wolfson Medical Center, Ds Holon 58100, Israel; 4 Gray Faculty of Medicine and Health Sciences, Tel Aviv University, Tel Aviv 6997801, Israel

**Keywords:** lung cancer, artificial intelligence, radiomics, personalized treatment, machine learning, deep learning, biomarkers

## Abstract

Lung cancer is the leading cause of cancer mortality globally, despite the advancements in screening and management. Survival rates for lung cancer remain suboptimal, largely due to late-stage diagnoses and tumor heterogeneity. Recent advancements in artificial intelligence and radiomics provide a promising outlook for lung cancer screening, diagnosis, personalized treatment, and prognosis. These advances use large-scale clinical and imaging datasets that help identify patterns and predictive features that may be missed by human interpretation. Artificial intelligence tools hold the potential to take clinical decision-making to another level, thus improving patient outcomes. This review summarizes current evidence on the applications, challenges, and future directions of artificial intelligence (AI) in lung cancer care, with an emphasis on early diagnosis and personalized treatment. We examine recent developments in AI-driven approaches, including machine learning and deep neural networks, applied to imaging (radiomics), histopathology, biomarker analysis, and multi-omic data integration. AI-based models demonstrate promising performance in early detection, risk stratification, molecular profiling (e.g., programmed death-ligand 1 (PD-L1) and epidermal growth factor receptor (EGFR) status), and outcome prediction. These tools may enhance diagnostic accuracy, optimize therapeutic decisions, and ultimately improve patient outcomes. However, significant challenges remain, including model heterogeneity, limited external validation, generalizability issues, and ethical concerns related to transparency and clinical accountability. AI holds transformative potential for lung cancer care but requires further validation, standardization, and integration into clinical workflows. Multicenter collaborations, regulatory frameworks, and explainable AI models will be essential for successful clinical adoption.

## 1. Introduction

Lung cancer continues to be the leading cause-related mortality worldwide, with 1.8 million reported deaths annually [1]. With the advancement in therapeutic approaches, particularly targeted therapies and immunotherapeutic treatments, overall survival rates remain poor. Late-stage diagnosis and substantial biological heterogeneity continue to limit treatment efficacy and long-term outcomes. This has highlighted the necessity for better stratification tools in favor of earlier diagnosis, prognosis, and individualized treatment planning.

**Figure 1:**
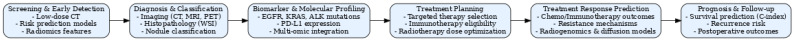
Applications of artificial intelligence across the lung cancer care continuum.

Artificial intelligence (AI) has shown potential for addressing a number of the issues present in lung cancer management. Using vast clinical, imaging, and molecular databases, AI programs have proven the potential for enhanced diagnostic precision, the identification of predictive biomarkers, and aid in therapeutic decision-making. It has the potential for pattern detection beyond human capabilities for both increased efficiency and clinical benefit. However, despite these promising developments, widespread clinical adoption of AI in oncology remains limited. Barriers such as model generalizability, transparency, and external validation continue to hinder integration into routine practice. Additionally, the complex molecular landscape of lung cancer—characterized by dynamic genomic alterations, tumor microenvironment heterogeneity, and adaptive resistance mechanisms—presents ongoing challenges for the development of robust and reliable predictive frameworks. This review aims to comprehensively examine the current state of AI applications in lung cancer, focusing on both diagnostic and therapeutic domains. It first outlines the key pathological and molecular features of lung cancer, establishing the foundation for understanding the clinical relevance of AI-based interventions. Subsequently, the review explores recent advancements in AI-assisted screening, subtype classification, predictive modeling for treatment response, and survival outcomes, while also addressing existing limitations and future research directions. An overview of AI applications across the lung cancer care continuum is illustrated in Figure 1.

## 2. Lung Cancer Definition

Lung cancer is the major cause of cancer death throughout the world, as stated by the World Health Organization (WHO) [1]. The disease arises from uncontrolled proliferation of respiratory tract epithelial cells, primarily driven by carcinogenic exposures such as tobacco smoke, environmental pollutants, and occupational hazards.

Histologically, lung cancer is broadly categorized into two major groups:

- Non-Small Cell Lung Cancer (NSCLC): Accounting for approximately 85% of all lung cancers, NSCLC encompasses three primary subtypes:
  - Adenocarcinoma: The most common subtype, particularly prevalent among females and non-smokers. It originates from glandular epithelial cells and typically presents in the peripheral regions of the lung.
  - Squamous Cell Carcinoma: Strongly associated with smoking, this subtype arises centrally near the bronchial airways.
  - Large Cell Carcinoma: A poorly differentiated and aggressive subtype characterized by rapid progression and unfavorable prognosis [2].
- Small Cell Lung Cancer (SCLC): Representing 10–15% of lung cancers, SCLC is distinguished by its neuroendocrine features, rapid proliferation, early metastasis, and strong association with tobacco use. Histologically, SCLC is composed of small cells with minimal cytoplasm and high mitotic activity [3].

The WHO categorization further delineates among these groups based on molecular and immunohistochemical markers to aid in personal treatment plans. With the increasing application of advanced molecular diagnostics, identification of subtypes to inform treatment has become increasingly necessary.

## 3. Pathophysiology

### 3.1. Genetic Alterations

Lung cancer arises through a multistep process involving the accumulation of genetic and epigenetic alterations in respiratory epithelial cells, often triggered by carcinogenic exposures such as tobacco smoke, industrial toxins, and environmental pollutants [4]. Over time, these insults drive the development of malignant clones characterized by dysregulated proliferation, impaired apoptosis, and altered differentiation. Key genetic alterations in lung cancer include mutations in both oncogenes and tumor suppressor genes. In non-small cell lung cancer (NSCLC), common driver mutations involve *EGFR*, *KRAS*, *ALK*, and *BRAF*, which activate oncogenic signaling pathways such as PI3K/AKT and RAS/MAPK, promoting uncontrolled cellular proliferation. In small cell lung cancer (SCLC), the hallmark molecular features are inactivating mutations in *TP53* and *RB1*, leading to profound cell cycle dysregulation and genomic instability [4]. These molecular alterations not only initiate tumorigenesis but also influence therapeutic sensitivity and the emergence of resistance. Beyond single-gene mutations, lung cancer exhibits considerable intra- and inter-tumoral heterogeneity. Next-generation sequencing has revealed the coexistence of multiple subclonal populations harboring distinct driver events within the same tumor. This heterogeneity complicates treatment responses and facilitates the development of resistance to targeted therapies. Consequently, lung cancer evolves as a dynamic mosaic of clonal expansions shaped by both genetic and microenvironmental pressures.

### 3.2. Epigenetic Modifications

Epigenetic alterations also play a critical role in lung carcinogenesis. Hypermethylation of tumor suppressor genes in the promoter regions of *CDKN2A*, *RASSF1A*, and *FHIT* results in their inactivation without affecting the underlying DNA sequence [5]. These are among the changes in the earliest phase of the neoplastic sequence and are thought to predispose for further genetic alterations. Global hypomethylation and remodeling of the chromatin also play a role in favoring the metastatic behavior and immune evasion.

MicroRNAs (miRNAs), a class of non-coding RNAs, also critically regulate gene expression post-transcriptionally and can function as either oncogenes or tumor suppressors. For instance, overexpression of *miR-21* promotes cell survival and chemoresistance, while decreased levels of the *let-7* family facilitate tumor proliferation [5]. Given their stability in circulation, miRNAs are being actively explored as non-invasive biomarkers for early detection, prognosis, and treatment monitoring in lung cancer.

### 3.3. Tumor Microenvironment

Another critical component of lung cancer pathophysiology consists of the tumor microenvironment (TME). Lung cancer does not develop in isolation but in a highly interactive stroma of immune cells, fibroblasts, endothelial cells, and extracellular matrix proteins. Tumor cells produce, interleukin-6, transforming growth factor beta, and vascular endothelial growth factor among others, that remodulate the nearby stroma, suppress immune surveillance, and cause angiogenesis [6]. These interactions set up a permissive environment for tumor growth as well as metastatic spreading. The pro-inflammatory state of the TME, often the result of chronic exposure to irritants such as cigarette smoke or particulate matter, also facilitates further tumorigenesis. Pro-inflammatory cytokines and reactive oxygen species are released by the infiltrating inflammation, leading to DNA damage and induction of a regenerative response. Chronic inflammation causes a vicious cycle of cell turnover and genetic instability, hence propelling the evolution of the tumor [6].

### 3.4. Immune Evasion and Tumor Cell Plasticity

Immune evasion represents a hallmark of lung cancer progression. Tumor cells exploit immune checkpoint pathways such as PD-1/PD-L1 to inhibit cytotoxic T-cell activity. Additionally, recruitment of regulatory T cells, myeloid-derived suppressor cells, and M2-polarized macrophages contributes to an immunosuppressive TME, promoting immune tolerance and resistance to immunotherapy [6]. Emerging evidence highlights tumor cell plasticity and stemness as additional contributors to lung cancer heterogeneity. Bronchoalveolar stem cells (BASCs) and alveolar type II cells have been implicated as cells of origin for lung adenocarcinoma, possessing both self-renewal and differentiation capacities that render them vulnerable to oncogenic transformation [7]. These progenitor populations may underlie the phenotypic diversity observed in advanced disease. In SCLC, bronchial epithelial progenitors of neuroendocrine origin—characterized by concurrent *TP53* and *RB1* inactivation—give rise to highly aggressive tumors with early metastatic spread [7]. Collectively, the pathophysiology of lung cancer reflects a complex interplay of genetic, epigenetic, cellular, and microenvironmental factors. This multifaceted biology not only drives tumor initiation and progression but also informs emerging strategies for early detection, therapeutic targeting, and precision medicine.

## 4. Artificial Intelligence

Artificial intelligence (AI) is the concept of computer algorithms having the ability to mimic human intelligence. Machine learning is a subset of AI that has the ability to learn without being explicitly programmed. This ability to learn has two phases: training and inference. A data set is given to the program in the training phase, from which the program can establish patterns. In the inference phase, these patterns are used on a new data set to perform certain tasks like prediction and identification. More complex ML algorithms appeared and included hierarchically organized layers, giving rise to deep learning models. This layer complexity allows the model to extract generic features from data sets without relying on pre-defined features [8].

Training frameworks for ML models depend on how much control of the input and output is involved. Supervised learning is the most rigid and safe framework, in which annotated input is provided and the output is controlled. This is the easiest way to train ML learning models. However, data curation is tedious, time consuming and requires significant expertise. In unsupervised learning, data is not annotated, allowing the model to discover patterns independently. Semi-supervised learning involves using partially labeled data. This is useful in cases in which large labeled data sets are difficult to obtain. Additionally, it minimizes the overfitting problem in supervised learning. That is when a model is trained too closely to fit the labeled training data set, it becomes unable to make decisions and conclusions on other data sets. Reinforcement learning models learn by receiving feedback and adjusting in a trial and error fashion. Transfer learning refers to reusing layers from a trained model and adjusting it to perform different tasks while ensemble models combine output from different models to perform tasks.

Many recent innovative approaches use hybrid and combination models that blur the line of supervised vs. unsupervised learning. The work flow of AI in medicine involves training AI models to learn and extract features from large data sets and perform tasks using the extracted features. For example, in radiomics, omic-like features are extracted from a large data set of medical images which can be used to perform tasks, or funneled into predictive models to make predictions. The most commonly used AI architectures in lung cancer research are summarized in Table 1.

## 5. AI in Lung Cancer Diagnosis

Artificial intelligence (AI) has emerged as a transformative force in lung cancer diagnosis. By addressing key limitations in early detection, image interpretation, and clinical decision-making, AI holds significant potential to enhance diagnostic accuracy and improve patient outcomes.

The researcher trains the AI models on an image data set, usually public data sets such as LUNA16 [9] or Lung Image Database Consortium Image Collection and Image Database Resource Initiative (LIDC-IDRI) [10], from which the AI model can detect features and patterns. The model then is tested on either the same or a different data set to check performance in the desired task.

AI algorithms have been studied on detection and classification of nodules from X-rays, CT, MRI, and PET scans, with most studies evaluating AI algorithms on CT images. Both machine learning and deep learning AI models have shown performance in nodule detection that is comparable to or surpasses radiologists [11,12,13,14]. However, the majority of research now focuses on DL algorithms due to their improved performance compared to classical ML algorithms. Convolutional neural networks (CNNs) and their derivatives (DCNN, U-net, 3D-CNNS) are particularly well-suited for the interpretation of radiologic images. CNN-based models trained on extensive image datasets, such as those from the National Lung Screening Trial (NLST), have achieved high sensitivity and specificity for the detection of pulmonary nodules on chest CT scans [13]. These models can identify subtle lesions that may be overlooked during manual interpretation, thus facilitating earlier detection and intervention. Deep convolutional neural networks (DCNN) demonstrated the highest accuracy in lung cancer detection compared with other CNN algorithms [15].

Transformers (e.g., vision transformers and SWIN) are a deep neural network algorithm that has different characteristics than CNNs like their self-attention property that allows them to understand complex patterns and global relationships [16]. They have the ability to process long-range discrepancies and spatial correlation, an advantage over CNNs that excel in short range discrepancies [17]. However, with insufficient quantities of data, they underperform compared to CNNs and they require significant computational power [18]. Recently, combination models were proposed to overcome the limitation of single architectures and offer improved performance compared to models relying on a single architecture. For example, Durgam et al. developed a model that combines vision transformer, U-net, CNN, deep LSTM (deep recurrent neural network architecture), and Generative Adversarial Networks (GANS) for the detection and segmentation of lung cancer in CT images [19]. Representative diagnostic AI models, their datasets, and performance are outlined in Table 2.

Beyond nodule detection, AI contributes to malignancy risk stratification and nodule classification through radiomics—the extraction of quantitative imaging features such as shape, texture, and intensity—and clinical data. These models capture information imperceptible to the human eye to differentiate benign and malignant lesions, thereby improving diagnostic precision and potentially reducing unnecessary invasive procedures [13,20]. For example, Zhu et al. developed the Cross-ViT model, a model combining CNN and Transformer, to classify lung nodules from CT images into benign and malignant nodules using the LUNA16 data set and compared their results with other classification models [21]. Another example is Lin et al.‘s model which used radiomics and clinical features to classify nodules by type (benign and malignant nodules), adenocarcinomapathological subtype, and Lung-RADS score [22].

AI-driven automated tumor segmentation enhances delineation of both the tumor and surrounding anatomy, which is crucial for precise treatment planning, including surgical resection and radiotherapy delivery. DL-based segmentation methods have demonstrated superior accuracy and efficiency compared to traditional techniques [23]. In this application, U-nets and GANS are commonly used. U-nets and their derivatives perform exceptionally well in tumor segmentation [24]. Importantly, AI models’ performance can depend on the imaging modality (X-ray, CT, MRI). For example, U-net derivatives and 3D CNN architecture perform exceptionally in volumetric scans like CT and MRI, while CNNs that used transfer learning frameworks excelled in cancer segmentation in X-ray images [25,26,27]. Generative AI is particularly useful because of its ability to generate 3D reconstruction of tumors. Limitations of generative AI being data discrepancies and high computational load [24].

Emerging evidence also suggests that AI may assist in differentiating histologic subtypes of non-small cell lung cancer (NSCLC) solely based on imaging features [20]. In pathology, AI-powered whole slide imaging (WSI) combined with DL models has facilitated the automated analysis of histologic specimens. These tools have demonstrated the capacity to reduce diagnostic variability, increase consistency, and expedite histopathological evaluations [28].

AI has also shown utility in individualized risk prediction and screening optimization. ML algorithms that integrate imaging data with demographic, behavioral, and clinical variables (e.g., age, smoking history, genetic markers) improve risk stratification and support targeted low-dose CT screening strategies. This is particularly valuable in regions with limited radiological expertise or constrained healthcare resources [28]. For example Dutta performed a comparative analysis of different ML algorithms and a neural network DL model in predicting lung cancer using symptomatic and lifestyle data. The deep learning model outperformed all the ML models [29].

**Table 2:** Key AI models in diagnosis of lung cancer, showing applications, model types, data used, and performance.

Study	Application	Model Architecture	Data Used	Performance	Key Strengths
Lee et al. [11]	Lung cancer detection on chest radiographs	Deep CNN (Lunit Insight)	Chest radiographs	AUC up to 0.99 Sensitivity 83–90% Specificity 97%	Large real-world cohort; outperformed radiologists in visible cancer detection
Zhu et al. [21]	Benign vs. malignant pulmonary nodule classification	Cross-ViT (CNN + Transformer fusion; optional SENet CNN branch)	LUNA16 (subset of LIDC-IDRI), CT images	Cross-ViT: ACC 91.04%, Cross-ViT (SENet): ACC 92.43%	Combines local CNN and global Transformer features via cross-fusion attention; SENet branch further boosts performance over state-of-the-art
Safarian et al. [28]	[^18^F]FDG PET/CT radiomics for lung cancer diagnosis, staging, and treatment planning	Multiple AI architectures (ML, DL, CNN, radiomics integration)	[^18^F]FDG PET/CT ± clinical, pathological, and molecular data	Diagnostic AUCs up to 0.94 Subtype differentiation AUC ~0.86; Staging AUC up to 0.88 Treatment response prediction ACC up to 93%	Comprehensive review of PET/CT radiomics in NSCLC; covers benign/malignant classification, subtype differentiation, molecular marker prediction, staging, prognosis, and therapy response; highlights integration of imaging with clinical and genomic data
Dutta et al. [29]	Lung cancer prediction from symptoms & lifestyle	ML (DT, RF, KNN, NB, AB, LR, SVM) vs. DL (Neural Networks, 1–3 hidden layers	Clinical & lifestyle data set (Kaggle)	Best NN (1 hidden layer, 800 epochs): ACC 92.86%; best ML (KNN): AUC 0.915	Rigorous preprocessing & feature selection

Despite these promising advancements, significant challenges persist. Many AI models are developed using highly curated datasets that may not reflect the heterogeneity of real-world populations, raising concerns about generalizability. For example, if the training data set happens to only contain large tumors, the model may have difficulties detecting smaller tumors and may miss them. Moreover, many studies do not validate their models on external data sets, feeding more into the overfitting problem. The absence of standardized validation frameworks, lack of algorithmic transparency, and variability in model performance limit widespread clinical adoption. Additionally, ethical concerns related to data privacy, bias, and clinical accountability must be addressed to ensure safe and equitable implementation of AI technologies [13,28]. In summary, AI has demonstrated substantial promise across multiple facets of lung cancer diagnosis, including imaging, pathology, and risk assessment. However, robust external validation, regulatory oversight, and clinical integration are essential to fully realize its potential in improving early detection and patient outcomes in lung cancer care.

## 6. AI in Personalized Treatment

Personalized treatment has become the cornerstone of non-small cell lung cancer (NSCLC) management, emphasizing accurate patient characterization to guide targeted therapeutic strategies [30]. The emergence of immunotherapy and targeted therapies has transformed lung cancer care, and numerous studies have explored the role of artificial intelligence (AI) in predicting molecular alterations, biomarker expression, and therapeutic response. Key applications of AI in personalized treatment for NSCLC, including biomarker prediction, treatment response, and prognosis, are summarized in Table 3.

### 6.1. AI for Biomarker Prediction

PD-L1 Expression: Programmed death-ligand 1 (PD-L1) expression serves as a key biomarker for selecting patients likely to benefit from immunotherapy [31]. Early and accurate prediction of PD-L1 status can improve clinical outcomes. AI models have been developed to non-invasively estimate PD-L1 expression in NSCLC patients. For example, Dia et al. applied six machine learning models to histological slides for predicting PD-L1 expression and progression-free survival (PFS). These models employed supervised learning classifiers, including random forest, support vector machines, and gradient boosting, trained on extracted histopathological features from whole-slide images. Feature selection was improved to lower dimensionality and make the results more generalizable [32]. Lu et al. integrated deep learning radiomics derived from CT images with clinical variables; the deep learning pipeline used a ResNet-50 backbone to obtain image features and logistic regression to sort them. Combining different types of data, like patient demographics and tumor staging, made it easier to tell the difference between groups [33]. While Wang et al. developed a multi-feature fusion model to classify PD-L1 expression levels into <1%, 1–49%, and ≥50% categories. Wang’s method used a multi-source feature fusion framework that combined texture-based biomarkers, clinical variables, and radiomics from PET/CT images. The model used a fully connected neural network to combine these, which made it more stable and better calibrated than unimodal models [34].

Several other studies employing PET/CT-based deep learning radiomics have reported area under the curve (AUC) values ranging from 0.6 to 0.96 [35,36,37,38,39]. Generally, multimodal models incorporating both radiomic and clinical features outperform radiomics-only approaches, suggesting the clinical utility of AI-based PD-L1 prediction as a complementary or surrogate tool to immunohistochemistry in therapeutic decision-making. EGFR Mutation Status: AI has also shown promising results in predicting EGFR mutation status, another key target for therapy in NSCLC. Cao et al. developed an MRI-based deep learning model that achieved an AUC exceeding 0.93 [40], while Jia et al. reported an AUC of 0.828, sensitivity of 74.7%, specificity of 79.1%, and accuracy of 77.2% using CT imaging combined with clinical features [41]. Multiple additional studies have consistently demonstrated high predictive performance, although further optimization and validation remain necessary [42,43,44,45,46,47,48].

### 6.2. AI in Predicting Treatment Response

Despite advancements in therapeutic options, a considerable proportion of lung cancer patients fail to derive significant clinical benefit [49]. Accurately predicting response to therapy is therefore essential for optimizing personalized treatment plans. Several AI models have been developed for this purpose, utilizing diverse data sources including CT imaging, pathology, and clinical data [50].

Other models have incorporated metabolomics, protein biomarkers, hematoxylin and eosin (H&E)-stained slides, and clinical variables to predict treatment efficacy, demonstrating encouraging results [51,52,53]. Additional studies have evaluated AI for predicting response to induction therapy in locally advanced NSCLC [54] and for estimating pathological complete response to neoadjuvant chemoimmunotherapy [55,56].

Zheng et al. performed a prospective study on 72 patients with stage III–IV NSCLC who had diffusion kurtosis imaging (DKI) and intravoxel incoherent motion (IVIM) performed prior to treatment using a 3.0 T MRI (Signa Premier, GE Healthcare, Chicago, IL, USA). Both single-slice and whole-tumor ROIs were used for histogram analysis. Treatment response was significantly predicted by the mean values of the perfusion fraction (f), true diffusion coefficient (Dslow), and ADC; the whole-tumor combined model (Dslow_mean, f_mean, f_90th) had an AUC of 0.968, and fmean had the highest single-parameter AUC (0.886). The whole-tumor method performed better than single-slice analysis, highlighting the importance of volumetric evaluation in identifying intratumoral heterogeneity and facilitating precise, non-invasive chemoimmunotherapy response prediction [46].

### 6.3. AI for Prognostic Assessment

Prognostic modeling is another area where AI offers significant potential. Wang et al. developed a deep learning model that predicted overall survival (OS) based on PD-L1 expression signatures, achieving a C-index—a measure of how well predicted risks map to true outcomes, in which values near 1.0 indicate better prognostication accuracy—of 0.89 [34]. Similarly, Mu et al. utilized ^18^F-FDG PET/CT-based deep learning models to predict durable clinical benefit, PFS, and OS in advanced NSCLC patients treated with immune checkpoint inhibitors, with C-indices ranging from 0.7 to 0.87 [35]. 

A separate study incorporating histopathology, clinical data, and genetic information achieved high predictive accuracy for both histologic subtype classification (AUC 0.9821) and survival at 1-, 2-, and 3-year intervals (AUCs 0.9134, 0.8706, and 0.8765, respectively) [57]. Furthermore, machine learning-based nomograms have shown strong prognostic performance in patients with malignant pleural effusion, achieving AUCs above 0.92 in both internal and external validation cohorts [58].

### 6.4. AI for Surgical and Radiotherapy Decision-Making

AI also plays a growing role in guiding complex clinical decisions regarding surgery and radiotherapy. For example, Subramanian et al. applied artificial neural networks to predict overall survival and disease progression in early-stage NSCLC patients treated with stereotactic body radiation therapy (SBRT) [59]. Multi-task deep learning networks have been developed to individualize radiotherapy doses and estimate treatment failure risk based on CT images and clinical factors [60].

### 6.5. AI for Postoperative Outcomes and Healthcare Utilization

Beyond oncologic outcomes, AI has been employed to predict postoperative mortality, functional disability, and hospital length of stay. For instance, Hashimoto et al. developed a machine learning model to predict in-hospital mortality and postoperative disability in elderly cancer patients, including those with lung cancer, achieving an AUC of 0.81 [61]. Another study demonstrated the ability of machine learning models to estimate hospital length of stay with high predictive performance [62].

**Table 3:** Key AI models in personalized treatment for NSCLC, showing applications, model types, data used, and performance.

Study	Application	Model Architecture	Data Used	Performance	Key Strengths
Lu et al. [33]	PD-L1 prediction	Deep learning radiomics (ResNet-50) + clinical fusion	CT + clinical data	AUC 0.91 (combined)	Multimodal fusion improves accuracy
Wang et al. [34]	PD-L1 prediction	Multi-source fusion (3D ResNet + radiomics + clinical)	CT + clinical data	AUC 0.950 (low), 0.934 (medium), 0.946 (high); C-index 0.89	Largest cohort; robust 3-class prediction; adds survival prognostics
Cao et al. [40]	EGFR mutation	Radiomics + random forest + clinical features	T1-CE & T2W MRI of brain metastases	AUC 0.931 (train), 0.892 (val) for radiomics; AUC 0.943 (train), 0.936 (val) for DL; AUC 0.938 overall	Non-invasive, high accuracy for BM genotyping, external validation, strong generalization
Jia et al. [41]	EGFR mutation	Deep Learning Radiological-Pathological-Clinical (DLRPC) model	Preoperative CT + sex + smoking history	AUC 0.828; sensitivity 60.6%, specificity 85.1%	Widely available CT; high specificity; practical in low-resource setting
Yang et al. [50]	Treatment response	Multisequence MRI deep learning radiomics (ResNet34) + radiomics features + stacking fusion	CT scans + H&E-stained biopsy images + clinical data	AUC = 0.8424; Accuracy = 79.8%; Sensitivity = 81.2%; Specificity = 76.4%; PPV = 92.1%, NPV = 61.3%	Multimodal feature fusion captures macro- and microstructure; high PPV supports treatment allocation
Zheng et al. [46]	Treatment response	Diffusion MRI models (IVIM & DKI) with histogram analysis (whole-tumor & single-slice ROI)	Pre-treatment 3.0T MRI (IVIM and DKI sequences)	Whole-tumor combined model (Dslow_mean, f_mean, f_90th) AUC = 0.968; best single parameter f_mean AUC = 0.886	Prospective design; volumetric analysis captures tumor heterogeneity better than single-slice; identifies f_mean as strong non-invasive imaging biomarker
Xie et al. [57]	Prognosis	Machine learning (XGBoost, RF, LightGBM, AdaBoost) and deep learning (MLP, TabNet, CNN)	Histopathological nuclear features + clinical data + genetic (mRNA, SNV, CNV)	Subtype: XGBoost AUC = 0.9821 (Acc = 94.0%); OS prediction: RF AUC = 0.9134 (1 yr), 0.8706 (2 yr), 0.8765 (3 yr	First to integrate nuclear morphology with clinical & genetic features; high accuracy across multiple outcomes; large, multi-institution data set

## 7. Current Limitation and Future Perspectives

Artificial intelligence (AI) holds significant promise for transforming lung cancer care; however, several critical limitations currently hinder its widespread clinical implementation. A major challenge lies in the lack of standardization across various aspects of data acquisition and model development. Variability in imaging protocols, histopathologic preparation, clinical data reporting, patient populations, and institutional practices compromises the consistency and generalizability of AI models. Moreover, studies often employ heterogeneous methodologies for model training, validation, and performance evaluation, which complicates inter-study comparisons and limits external applicability.

Replication studies are necessary to ascertain the reproducibility of AI models and external validation. For example, Stüber et al. tried to replicate Zhao et al.’s machine learning algorithm to infer PD-L1 from PET/CT images, but they were unable to replicate the same, which cast doubt on the robustness of the model [63,64]. Additionally, many researchers do not include external validation cohorts or do not compare their models against seasoned radiologists, limiting their clinical reliability in the process.

Another significant limitation is that several AI systems, particularly deep learning models that function as “black boxes” are not easy to interpret. The inability to explain how these models make decisions internally lowers clinicians’ trust and slows their adoption in practice. Future studies should prioritize the development of explainable AI (XAI) models to increase transparency, gain clinician trust, and enhance decision support making [62,65].

Economic considerations also represent a substantial barrier to AI integration. Although AI has demonstrated potential cost savings in select clinical scenarios [66], comprehensive cost-effectiveness analyses remain scarce [67]. Detailed evaluations of implementation costs, including staff training, infrastructure requirements, and long-term maintenance, are necessary to inform healthcare resource allocation and guide institutional planning [67,68]. Many healthcare systems currently lack the financial and human resources required to integrate, operate, and sustain AI-based systems at scale [69].

Both clinician and patient acceptance are critical factors influencing AI adoption. Clinician trust may be undermined by concerns about algorithmic bias, particularly in underserved populations where AI models may perform suboptimally due to limited training data diversity [69]. Legal liability, responsibility for clinical outcomes, and accountability for AI-driven decisions also pose ethical and medico-legal challenges that may deter clinician utilization [70]. From the patient perspective, issues related to loss of human interaction, data privacy, safety, and algorithmic reliability may contribute to skepticism regarding AI integration in healthcare [71]

Several reporting and regulatory frameworks have been introduced to improve the transparency and quality of AI research, including the CONSORT-AI and SPIRIT-AI guidelines [72,73]. However, adherence to these standards remains limited [74], and few studies fully comply with the American Society of Clinical Oncology’s transparency principles for AI applications [75]. Moreover, while many jurisdictions regulate AI under the Software as a Medical Device (SaMD) framework, significant regulatory gaps persist, particularly concerning algorithmic updates, adaptive learning models, and international harmonization [76,77].

To advance AI toward routine clinical application in lung cancer care, future research must address these limitations through multicenter collaborations and the development of large, standardized, and diverse datasets [78]. Rigorous methodological standardization will improve reproducibility and facilitate cross-study comparisons [79,80]. Furthermore, robust external validation strategies, ongoing model monitoring, and regulatory oversight will be essential to ensure the safety, reliability, and clinical utility of AI technologies.

## 8. Conclusions

Artificial intelligence holds substantial potential to address many of the persistent challenges in lung cancer care. Recent studies have demonstrated encouraging results in the application of AI for early diagnosis, molecular characterization, risk stratification, treatment response prediction, and prognostic assessment. While these advancements are promising, significant limitations—including issues of standardization, generalizability, transparency, and clinical integration—remain obstacles to widespread adoption. Ongoing research, supported by rigorous validation, multicenter collaborations, and adherence to emerging guidelines, will be essential to overcome these barriers. As AI technologies continue to mature, they are poised to play a transformative role in shaping the future of personalized lung cancer management and improving patient outcomes.

To facilitate clinical implementation, external validation and adherence to international reporting guidelines such as TRIPOD, CONSORT-AI, and SPIRIT-AI are essential. Explainable AI systems should be emphasized with the intent of enhancing clarity and clinician confidence. AI models should adopt multimodal approaches that include imaging, clinical, and molecular data, as they have demonstrated superiority to single-modality models. Importantly, AI technologies should serve to complement, not to replace, clinician work flow. Future initiatives should include studies of cost-effectiveness to insure accessibility and sustainability across varying healthcare systems.

## Figures and Tables

**Table 1:** Common machine learning and deep learning architectures used in lung cancer research.

AI Model	Common Architectures
Machine Learning	Logistic regression Entropy degradation method (EDM) K-Nearest Neighbors (KNN) Gradient Boosting (XGBoost, LightGBM) Support vector machine (SVM) Partially observable Markov decision Process (POMDP) Random forest neural network (RFNN)
Deep Learning	Artificial Neural Networks (ANN) Convolutional neural networks (CNNs) U-Net 3D CNN Recurrent Neural Network (RNN) Transformer Long Short-Term Memory (LSTM) Generative adversarial networks (GANs)

## Data Availability

No new data were created or analyzed in this study.

## Abbreviations

The following abbreviations are used in this manuscript:

AI	Artificial Intelligence
ML	Machine Learning
DL	Deep Learning
CNN	Convolutional Neural Network
DCNN	Deep Convolutional Neural Network
U-Net	Convolutional Neural Network architecture for image segmentation
RNN	Recurrent Neural Network
LSTM	Long Short-Term Memory
GAN	Generative Adversarial Network
ANN	Artificial Neural Network
KNN	K-Nearest Neighbors
SVM	Support Vector Machine
RFNN	Random Forest Neural Network
POMDP	Partially Observable Markov Decision Process
EDM	Entropy Degradation Method
AUC	Area Under the Curve
C-index	Concordance Index
NSCLC	Non-Small Cell Lung Cancer
SCLC	Small Cell Lung Cancer
PD-L1	Programmed Death-Ligand 1
EGFR	Epidermal Growth Factor Receptor
KRAS	Kirsten Rat Sarcoma Viral Oncogene Homolog
ALK	Anaplastic Lymphoma Kinase
BRAF	v-Raf Murine Sarcoma Viral Oncogene Homolog B1
TP53	Tumor Protein p53
RB1	Retinoblastoma 1
CDKN2A	Cyclin Dependent Kinase Inhibitor 2A
RASSF1A	Ras Association Domain Family Member 1A
FHIT	Fragile Histidine Triad Protein
miRNA	MicroRNA
CT	Computed Tomography
PET	Positron Emission Tomography
MRI	Magnetic Resonance Imaging
[^18^F]FDG	Fluorodeoxyglucose (radiotracer)
IVIM	Intravoxel Incoherent Motion
DKI	Diffusion Kurtosis Imaging
H&E	Hematoxylin and Eosin
PFS	Progression-Free Survival
OS	Overall Survival
SaMD	Software as a Medical Device
CONSORT-AI	Consolidated Standards of Reporting Trials–Artificial Intelligence
SPIRIT-AI	Standard Protocol Items: Recommendations for Interventional Trials–Artificial Intelligence
TRIPOD	Transparent Reporting of a Multivariable Prediction Model for Individual Prognosis or Diagnosis
